# Expression of serum amyloid A in uterine cervical cancer

**DOI:** 10.1186/1746-1596-9-16

**Published:** 2014-01-21

**Authors:** Yanjie Ren, He Wang, Donghao Lu, Xiaoyan Xie, Xinlian Chen, Jing Peng, Qian Hu, Gang Shi, Shanling Liu

**Affiliations:** 1 Department of Obstetrics&Gynecology, West China Second University Hospital, Sichuan University, No. 20, 3rd Section of Ren Min Nan Road, Chengdu 610041, China; 2 Laboratory of Genetics, West China Institute of Women and Children's Health, West China Second University Hospital, Sichuan University, Chengdu 610041, China; 3 Key Laboratory of Obstetric&Gynecologic and Pediatric Diseases and Birth Defects of Ministry of Education, Chengdu 610041, China; 4 Center of Prenatal Diagnosis of Sichuan Province, West China Second University Hospital, Sichuan University, Chengdu 610041, China; 5 Laboratory of Cell and Gene Therapy, West China Institute of Women and Children's Health, West China Second University Hospital, Sichuan University, Chengdu 610041, China; 6 Department of Medical Epidemiology and Biostatistics, Karolinska Institutet, Stockholm SE 171-77, Sweden

**Keywords:** Uterine cervical carcinoma, Serum amyloid A, Tumor marker

## Abstract

**Background:**

As an acute-phase protein, serum amyloid A (SAA) is expressed primarily in the liver. However, its expression in extrahepatic tissues, especially in tumor tissues, was also demonstrated recently. In our study, we investigated the expression of SAA in uterine cervical carcinomas, and our results suggested its potential as a serum biomarker.

**Methods:**

Quantitative real-time polymerase chain reaction (RT-PCR), immunohistochemistry (IHC) and enzyme-linked immunosorbent assay (ELISA) were used to evaluate the SAA gene and protein expression levels in the tissues and sera of patients with non-neoplastic lesions (NNLs), cervical intraepithelial neoplasia (CIN) and cervical carcinoma (CC).

**Results:**

Compared with NNLs, the SAA gene (*SAA1* and *SAA4*) expression levels were significantly higher in uterine CC (mean copy numbers: 138.7 vs. 5.01, P < 0.000; and 1.8 vs. 0.079, P = 0.001, respectively) by real-time PCR. IHC revealed cytoplasmic SAA protein staining in tissues from adenocarcinoma and squamous cell carcinoma of the cervix. The median serum concentrations (μg/ml) of SAA were 6.02 in patients with NNLs and 10.98 in patients with CIN (P = 0.31). In contrast, the median serum SAA concentration was 23.7 μg/ml in uterine CC patients, which was significantly higher than the SAA concentrations of the NNL group (P = 0.002) and the CIN group (P = 0.024).

**Conclusions:**

Our data suggested that SAA might be a uterine CC cell product. High SAA concentrations in the serum of CC patients may have a role in monitoring disease occurrence and could have therapeutic applications.

**Virtual slides:**

The virtual slide(s) for this article can be found here: http://www.diagnosticpathology.diagnomx.eu/vs/1433263219102962.

## Introduction

Each year, more than 530,000 women develop cervical carcinoma (CC), and 270,000 die from this disease globally [1], which makes CC the third most common and the fifth most lethal cancer worldwide. Based on both clinical and histopathological variables, uterine cervical diseases are categorized into three groups: non neoplastic, intraepithelial neoplasia and carcinoma. Carcinoma is further classified into two main subtypes: cervical adenocarcinomas and squamous cell carcinoma (SCC). The survival rate for patients with CC, similar to many other cancers, is also related to early disease detection. The patient survival rate depends significantly on the clinical (FIGO: International Federation of Gynecology and Obstetrics) stage and is as high as 90% (good) when the disease is diagnosed early and as low as 15% (poor) when the diagnosis is delayed [2-4]. Therefore, early diagnosis and treatment could significantly increase the survival rates of CC patients and their quality of life. As oncogenic human papillomavirus (HPV) can be detected in nearly all CC patients, the role of HPV in the progression of CC has been determined. Over the course of years, only a small proportion of women with a chronic HPV infection will develop cancer, which may underline the potential diverse patterns of HPV infection. Therefore, although HPV DNA testing appears to be sensitive, it lacks specificity [5-7], and a recent report indicated that some types of CC, such as minimal deviation adenocarcinoma, were not associated with high-risk HPV [8]. Thus, in addition to HPV DNA testing, the discovery of novel diagnostic and therapeutic markers against this malignancy should be prioritized.

Serum amyloid A (SAA) is a positive acute-phase protein with four different isotypes in humans. SAA is generated primarily by the liver in response to trauma, infection, inflammation, and even neoplastic stimuli [9]. SAA1 and SAA2 each consist of 122 amino acids, including signal peptide sequences, and share more than 90% amino acid identity [10]. In addition, SAA3 is a pseudogene, and SAA4 is constitutively expressed at a constant level and is thus known as cSAA. The acute-phase SAAs (aSAA), SAA1 and SAA2, increase in concentration approximately several hundred-fold in response to inflammatory stimuli [11,12]. However, recent studies have revealed other major sources of SAA outside of the liver: specifically, cancer tissues of the esophagus [13], lung [14], pancreas [15], ovary [16], and uterine endometrium [5]. Moreover, recent data suggest that A-SAA, by activating the transcriptional factor nuclear factor kappa-B (NFκB) [17-21] and inducing the expression of matrix metalloproteinases (MMPs) [22-24], could considerably influence carcinogenesis. NFκB is a transcriptional factor for many functionally heterogeneous genes, notably the cytokine genes and anti-apoptotic genes. The activation of these genes could suppress apoptosis [25]. Additionally, a number of mechanisms have shown that MPPs could stimulate angiogenesis and activate cell migration.

To the best of our current knowledge, the localized expression of SAA in CC has not yet been reported. In this study, using RT-PCR, immunohistochemistry (IHC), and ELISA, we aimed to examine SAA expression and secretion in human CC.

## Materials and methods

### Tissues and serum

A total of 31 cervical tissue samples and 138 serum specimens obtained from West China Second University Hospital, Sichuan University from March 2011 to September 2012 were analyzed and segregated into the following diagnostic groups: non-neoplastic lesion (NNL), cervical intraepithelial neoplasia (CIN 1, CIN 2, and CIN 3), and invasive neoplasia (FIGO 2009 stages I and II) (detailed in Table 1). Freshly frozen biopsies were assessed using quantitative real-time polymerase chain reaction (RT-PCR) for SAA expression and were derived from primary specimens staged according to the FIGO surgical staging system, including 21 CC and 10 NNL cervical control samples obtained from hysterectomy specimens from women of similar ages [respective mean ages of 47.5 ± 3.1 (SD) and 42.7 ± 3.2 (SD) years old, respective ranges of 37–64 and 31–64 years old]. Formalin-fixed and paraffin-embedded cervical tissues were submitted to IHC testing, including 16 CC [4 adenocarcinoma and 12 squamous cell carcinoma, mean age 49.8 ± 4.3 (SD) years old, range 34–65 years old] and 5 NNL cervical control samples [5 uterine fibroids, mean age 43.6 ± 4.5 years old, range 31–59 years old]. The demographics and histological findings of the CC patients were graded and staged; the types of sections included in this study are detailed in Table 1. Serum samples measured by ELISA included the following: 32 women with NNL diseases [mean age 43.4 ± 8.4 (SD) years old, range 34–59 years old], 34 women with CIN [mean age 40.3 ± 7.5 (SD) years old, range 31–57 years old], and 72 women with histologically proven primary uterine cervical carcinoma [mean age 48.4 ± 8.7 (SD) years old, range 35–63 years old] (detailed in Table 1). The inclusion and exclusion criteria of the trial are detailed in Table 2. All samples tested were derived from patients who had provided written informed consent, and the study was approved by the ethics committee of West China Second Hospital of Sichuan University.

**Table 1:** Clinicopathological characteristics of patients enrolled in this study

**Characteristics**	**RT-PCR**		**IHC**		**ELISA**	
	**Number**	**Percentage (%)**	**Number**	**Percentage (%)**	**Number**	**Percentage (%)**
**Histological type**						
Adenocarcinoma	2	6.4	4	19.0	6	4.3
Squamous cell carcinoma	19	61.3	12	57.1	66	47.8
CIN		0		0	34	24.6
Uterine fibroids	10	32.3	5	23.9	21	15.2
Endometrial polyps		0		0	5	3.6
Ovarian cysts		0		0	6	4.3
**Grade of tumor**						
Well differentiated	2	9.5	4	25	19	26.4
Moderately differentiated	2	9.5	5	31.3	11	15.3
Poorly differentiated	17	81.0	7	43.7	42	58.3
**Histological stage**						
CINI					1	2.9
CINII					5	14.7
CINIII					28	82.4
**FIGO stage (Pre-NAC)**						
Ia		0	2	12.5	3	4.1
Ib	12	57.1	6	37.5	30	41.7
IIa	6	28.6	5	31.3	30	41.7
IIb	3	14.3	3	18.7	9	12.5
**Lymph node metastasis**						
Negative	10	47.6	5	31.3	21	29.2
Positive	11	52.4	11	68.7	51	70.8

NAC, Indicate neoadjuvant chemotherapy; CIN, Cervical intraepithelial neoplasias; SD, Standard deviation.

**Table 2:** Criteria for inclusion and exclusion in this trial

**Inclusion criteria**	**Exclusion criteria**
Newly diagnosed, pathologically confirmed diagnosis of CC	Chemotherapy, biologic therapy or any other investigational drug for any reason within 28 days prior to sampling
No previous chemotherapy or radiation therapy	Major surgical procedure, or significant traumatic injury within 28 days prior to sampling
No concurrent disease(s)	Pregnancy
Signed informed client consent	

### RNA extraction and quantitative real-time PCR

Total RNA from all primary snap-frozen samples, including the 21 CCs and 10 NNL cervical sample controls, was isolated using TRIzol Reagent (Invitrogen, Carlsbad, CA) according to the manufacturer’s instructions. The purity and amount of RNA were determined by measuring the ODs at a ratio of 260 to 280 nm. cDNA was reverse-transcribed from 1 μg of total RNA using the PrimeScript® RT reagent kit with gDNA Eraser (Perfect Real-Time) (Takara Biotechnology, Dalian, China). Quantitative PCR was performed with a CFX96 Touch Real-Time PCR Detection System using the manufacturer’s recommended protocol (Bio-Rad Laboratories, Hercules, CA) to evaluate SAA expression in all the samples. Each reaction was run in triplicate. Briefly, 2 μl of each cDNA sample (from a 20-μl total volume) was amplified using SsoFast EvaGreen® Supermix (BIO-RAD) to produce PCR products specific for SAA. The primer sequences specific for the two known human SAA genes, SAA1 and SAA4, were previously described in [26]. β-2 M served as a housekeeping gene for standardization. The negative control included replacement of the cDNA with H_2_O in the PCR.

### SAA immunostaining of formalin-fixed tumor tissues

Formalin-fixed paraffin-embedded NNL cervical control tissues and tumor tissues were evaluated using standard immunohistochemical staining for SAA expression. Study blocks (21 samples, including 16 CCs and 5 NNL cervical control samples) were selected after histopathologic review by surgical pathologists. The most representative block was selected for each specimen. Briefly, deparaffinized and rehydrated sections were treated according to the manufacturer’s instructions (DAKO Corporation; Carpinteria, CA). Eighty microliters of ready-to-use antibodies (clone mc1, DAKO) was used in each slice, which was then incubated for 2 hours at room temperature. The slides were washed with PBS and then incubated (1 hour at room temperature) with goat anti-mouse biotinylated secondary antibody (ZSGB-BIO, Beijing, China). The colorimetric readout was developed in 8 minutes using a DAB kit (ZSGB-BIO), followed by counterstaining with hematoxylin. The anti-SAA monoclonal antibody used (clone mc1, DAKO) was directed against amyloid fibril protein AA. The specificity of this antibody was previously described in [14]. For the negative control, the primary antibodies were replaced with PBS. Liver sections were used as positive controls.

### Measurement of SAA concentrations in serum samples

The SAA serum levels were determined using an SAA1-specific ELISA kit (Invitrogen). Samples collected before surgery were centrifuged at 1,600 × *g* for 15 min within 2 hours of collection and stored at -80°C until analysis. All the serum samples were thawed on ice for 40 minutes, allowed to reach room temperature, and diluted 200-fold with the standard diluent buffer provided in the kit. ELISAs were then performed according to the manufacturer’s instructions. After adding the Stop Solution to each well, the solution color changed from blue to yellow. The absorbance of each well was read on a Bio-Rad Model-680 Instrument (Bio-Rad Laboratories, Hercules, CA) at 450 nm to determine the SAA concentrations. The plate was read within 30 minutes after adding the Stop Solution. All specimens were tested in replicate wells. The results were reported as the means of the replicates. A standard curve was run in each assay.

### Statistical analysis

For quantitative real-time PCR, the fold change of mRNA was calculated using the 2^ΔΔCt^ method (ΔCt = the difference in threshold cycles for the target and β-2 M), with normalization to the level of β-2 M, and the results were compared for differences using the equal-variance t-test for the CC samples versus the NNL cervical samples. All images were captured using a Nikon Eclipse 55i microscope (Minato-ku, Japan), and the different expression levels among cervical tissues were analyzed using IHC. SAA serum concentrations among the different groups of patients (i.e., NNL diseases, cervical intraepithelial neoplasias, and cervical carcinomas, with different degrees of differentiation) were calculated from standard curves and summarized as medians and ranges. The differences were compared using the Wilcoxon-Kruskal-Wallis Test. SPSS 16 (SPSS Inc., Chicago, IL) for the statistical analysis. A 5% significance level was used for all statistical comparisons.

## Results

### SAA expression in snap-frozen cervical carcinoma tissues by quantitative real-time PCR

**Figure 1:**
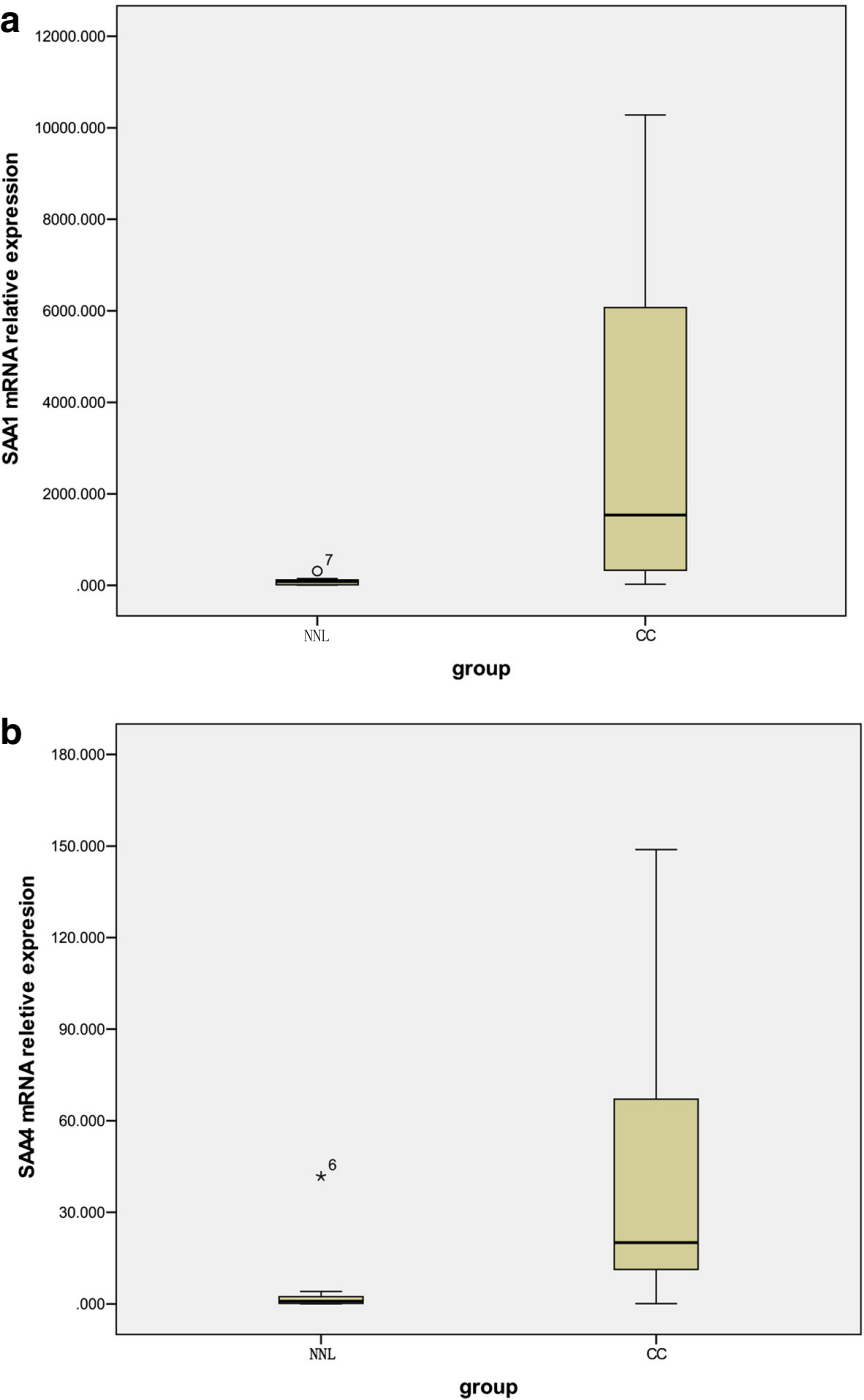
**mRNA expression of SAA1 and SAA4 in freshly frozen biopsies.** Expression of SAA1 **(a)** and SAA4 **(b)** by quantitative real-time polymerase chain reaction (RT-PCR) in 10 non neoplastic lesion cervical control samples and 21 cervical carcinoma freshly frozen biopsies are shown. The vertical axis represents the relative values of threshold cycle corrected with β2M (2^-ΔCt^). Values are means ± SD of triplicate measurement.

**Figure 2:**
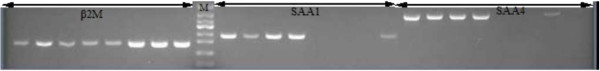
**SAA mRNA expression by electrophoresis.** Representive SAA1 and SAA4 PCR fragments were analyzed on a 2% agarose gel. In each 8 lanes, the first four were derived from different cervical carcinoma tissues and the rest were from non neoplastic lesion cervical tissues. Markers of a DNA ladder (50-bp steps) are shown in lane M. Using primers for SAA1, SAA4, and the control gene β-2 M analysis 10 benign cervical and 21 cervical carcinomas (2 adenocarcinomas and 19 squamous cell carcinomas) patients by RT-PCR.

The expression of *SAA1* was remarkably up-regulated in CC tissues compared with NNL cervical tissues. SAA4 had an expression pattern similar to that of *SAA1* (Figure 1; Table 3). The relative threshold cycle (ΔCt) values of *SAA1* and *SAA4* in the NNL cervical control samples were 7.64 ± 2.02 and 13.63 ± 3.11 (mean ± standard error), respectively, and the ΔCt values in the CC samples were 2.85 ± 3.02 and 9.12 ± 3.05 (mean ± standard error), respectively (Table 3). Using the 2^ΔΔCt^ method, the *SAA1* and *SAA4* expression levels in the CC samples were 27.67 (Table 3, P < 0.000) and 22.87 (Table 3, P = 0.001) times higher, respectively, than in NNL control tissues. Figure 2 shows weak to barely detectable *SAA1* and *SAA4* gene expression in NNL cervical tissues and strong expression in carcinoma tissues by electrophoresis on a 2% agarose gel. The control β2M gene was expressed at comparable levels in all samples.

**Table 3:** SAA mRNA expression by RT-PCR

**Variable**	**No**	**ΔCt (Mean ± SD)**	
		*SAA1*	*SAA4*
NNL tissues	10	7.64 ± 2.02	13.63 ± 3.11
CC tissues	21	2.85 ± 3.02	9.12 ± 3.05
ΔΔCt		4.79 (P < 0.000)	4.51 (P = 0.001)
2^ΔΔCt^		27.67	22.78

SD, Standard deviation; NNL, Non neoplastic lesion; CC, Cervical carcinoma; ΔCt, The difference in threshold cycles for the target gene; ΔΔCt, The difference in ΔCt values for the target gene.

### SAA expression by immunohistochemistry in cervical carcinoma tissues

**Figure 3:**
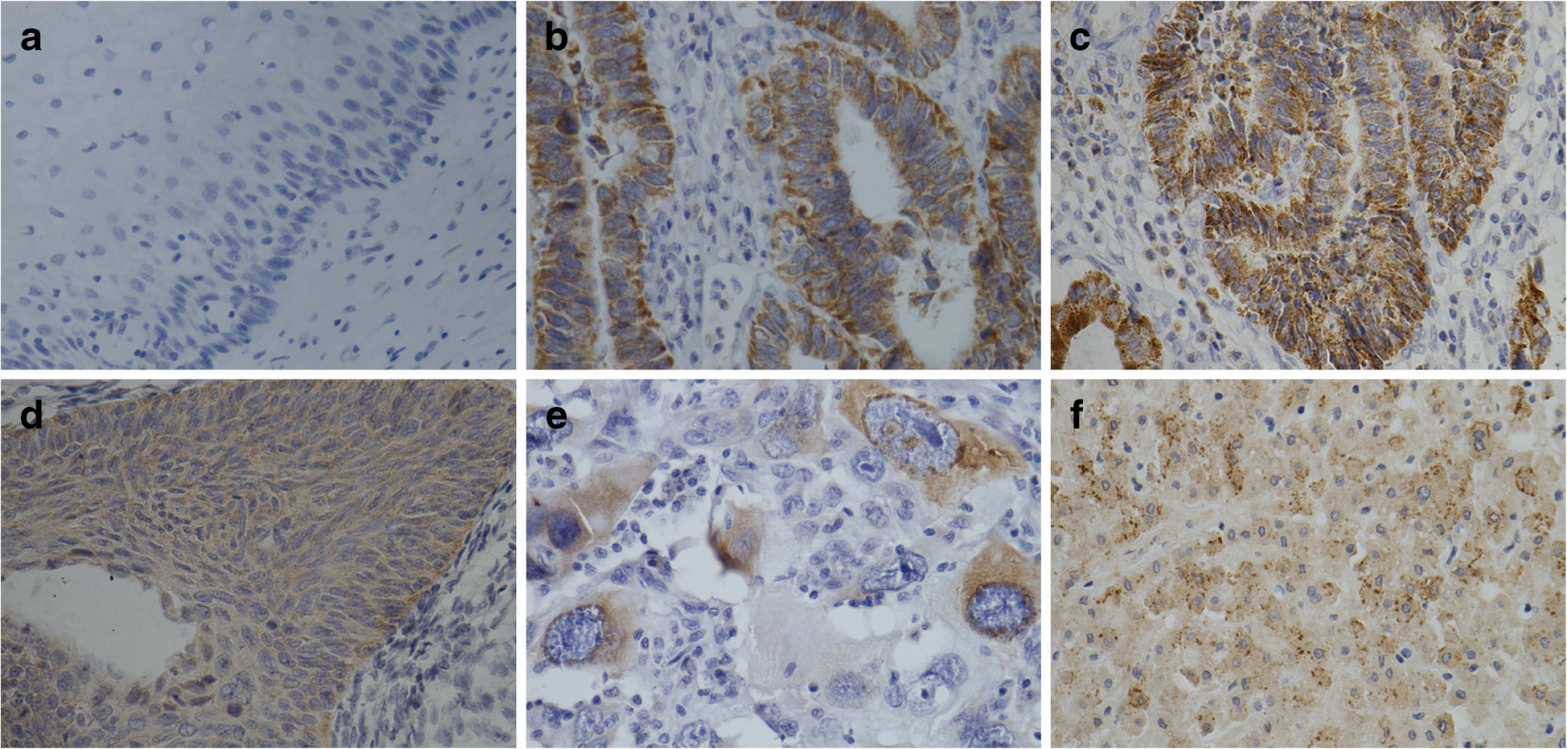
**SAA protein expression by IHC.** IHC demonstrating SAA protein expression in cervical carcinomas. The sections were immunostained with monoclonal anti-SAA antibodies. The reddish-brown staining represents positive SAA protein signal; counterstaining is light blue. **(a)** Non neoplastic lesion cervical tissue. The tumoral epithelium stained moderate to strong and focal. **(b, c)** Adenocarcinoma. The stage I cervical squamous carcinoma tissues stained moderate and focal. **(d)** and diffuse **(e)**.Stage II. Strong cytoplasmic SAA positivity was evident in the positive control **(f)** Liver. Original magnification x400 **(a-f)**.

Figure 3 shows positive cytoplasmic SAA protein expression levels in all cervical carcinoma tissues, as detected by IHC. In contrast, no SAA positivity was detected in NNL cervical tissues (Figure 3). However, there were differences in the staining patterns between squamous cell carcinoma and adenocarcinoma and between stages I and II. Normal liver tissue (i.e., the positive control) was strongly positive for SAA expression (Figure 3).

### Serum SAA concentrations in cervical carcinoma, cervical intraepithelial neoplasia, and benign disease patients

**Figure 4:**
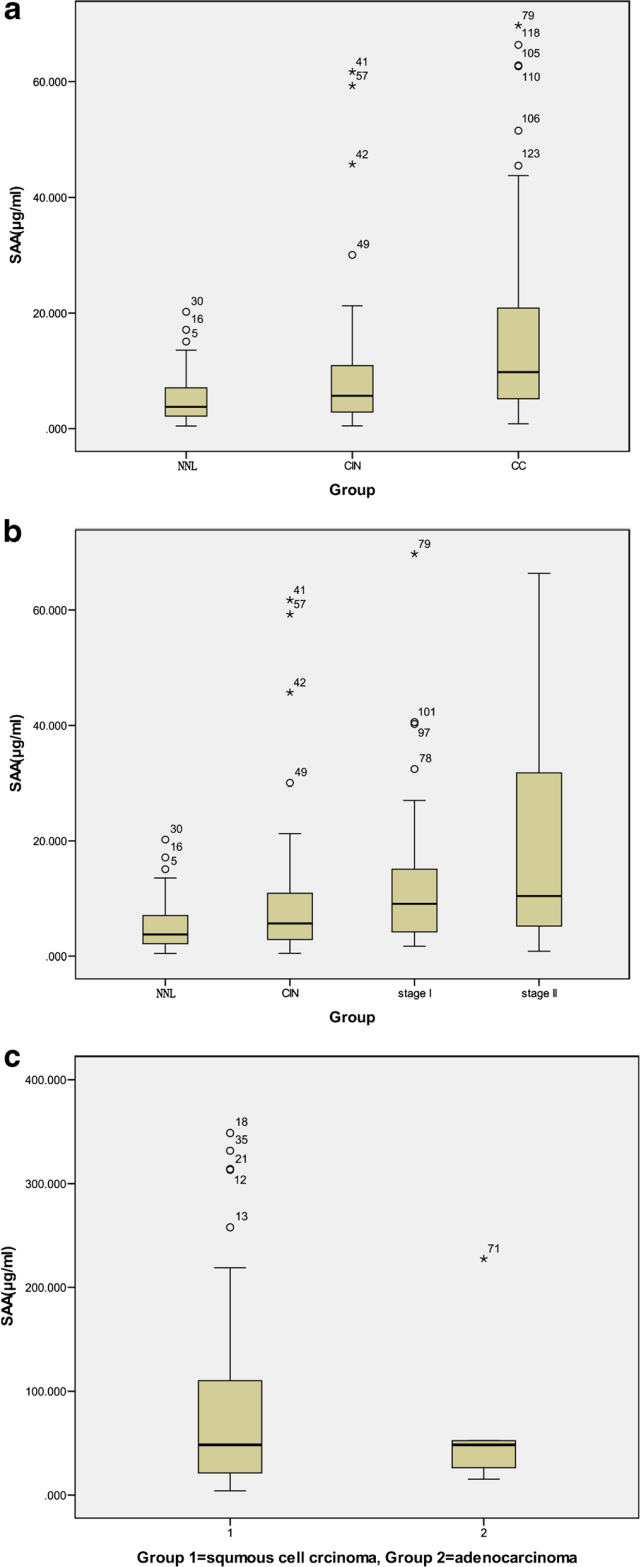
**SAA serum levels by ELISA. (a)** SAA serum levels in patients with cervical carcinoma. Serum levels of SAA were measured in patients with cervical pathologies classified as follows: (1) Non neoplastic lesion gynecologic diseases (n32), (2) cervical intraepithelial dysplasia (n35), (3) cervical carcinoma (n72). **(b)** SAA levels in cervical carcinoma with different degrees of stage (ie, 34 stages I, 37 stages II) are shown. Data are presented as mean ± standard deviation of mean. **(c)** SAA levels in cervical carcinoma with different histological types (ie, 66 squamous cell carcinomas, 6 adenocarcinomas) are shown.

Our data demonstrate that the SAA serum expression levels were comparable between patients with CIN and NNL gynecologic diseases (Figure 4; Table 4). The median SAA serum levels were 9 for the 32 control patients with NNL gynecologic disease and 14 for the 35 patients with CIN; their distributions were not significantly different (P = 0.31, Table 4 and Figure 4a). In contrast, the serum SAA values from CC patients were significantly higher than the values in the NNL group (P = 0.002) and in the CIN group (P = 0.024, Table 4 and Figure 4a). However, no differences were observed between different clinical stages (stages I and II, P = 0.483, Table 4 and Figure 4b) and histological types (adenocarcinoma and squamous cell carcinoma, P = 0.626, Table 4 and Figure 4c).

**Table 4:** Serum SAA Levels in Benign Disease, CIN, and CC Patients

**Group**	**Number**	**Median**
NNL	32	9.2
CIN	34	14.1
Stage I	33	22.7
Stage II	39	24.3
Adenocarcinoma	6	20.2
Squamous cell carcinoma	66	25.9
**Pairwise Comparison**		**P**
NNL versus CIN		0.31
NNL versus stage I		0.002
CIN versus stage I		0.024
Stage I versus stage II		0.483
Adenocarcinoma and squamous cell carcinoma		0.626

NNL Indicates non-neoplastic lesion; CIN Indicates cervical intraepithelial neoplasias; CC Cervical carcinoma; SD, Standard deviation; P Values are from 2-sided Wilcoxon-Kruskal-Wallis tests for the indicated comparisons.

## Discussion

In this study, using immunohistochemistry and real-time PCR analyses, we demonstrated the local over-expression of SAA at the protein and mRNA levels in human uterine cervical carcinomas, which may suggest that the tumor cells are the predominant source of SAA in cancer patients. In addition, we evaluated SAA levels in 138 serum samples derived from patients with NNL diseases of the cervix, CIN (precursor of cancer), and uterine cervical carcinomas and found that the circulating levels of SAA gradually increased with the carcinogenesis and progression of cervical cancer. Our findings suggest that there is a critical role for SAA in cervical carcinogenesis and may provide a promising target for future clinical therapy and postoperative surveillance.

Our findings indicate that the elevated SAA levels in local tissues and sera were due to its expression and secretion from CC cells. First, we reported a high SAA gene expression level in CC cases. By real-time PCR, the SAA1 and SAA4 mRNA expression levels were significantly higher in CC tissues than in NNL cervical tissues, with 27.7- and 22.8-fold higher copy numbers, respectively. Additionally, cytoplasmic positivity for SAA was detected by IHC in all CC-derived tissues, which verified that the presence of SAA mRNA is accompanied by SAA protein synthesis. Furthermore, in addition to the high levels of SAA gene expression, we detected high levels of circulating SAA. Additionally, in the limited number of patients undergoing chemotherapy, the serum levels of SAA were low, which could indirectly confirm that CC cells could secrete and express SAA.

Moreover, we also found that SAA expression was associated with CC progression. Notably, although there were no significant differences between the serum levels of the patients harboring NNL gynecological diseases and CIN and patients with stage I and II CC, the mean SAA serum levels of these groups were gradually increased with disease progression (Figure 4b; Table 4), with the highest serum levels in the stage II group. Cocco [5] and Urieli-Shoval [16] recently reported on SAA expression in two major gynecological cancers, endometrial endometrioid carcinoma and ovarian epithelial tumor, respectively. Using in situ hybridization and IHC, they demonstrated local and differential expression of SAA in human endometrial carcinoma and ovarian epithelial tumors compared with normal endometrial and ovarian epithelial tissues. Furthermore, they demonstrated that there was progressively higher SAA positivity through the different stages of dysplasia to overt carcinoma. Although the biological importance of SAA in cancer patients is not well understood, these findings in human endometrial endometrioid carcinoma and ovarian epithelial tumors, combined with our results in uterine cervical carcinoma, appear to suggest a novel role for SAA autocrine production in gynecological tumorigenesis and progression.

Several studies have proposed that SAA protein functions are relevant to tumor cell invasion and metastasis. First, SAA may influence carcinogenesis. As mentioned above, SAA could activate the transcriptional factor NFκB, which could suppress apoptosis. Given that inflammation has recently been proposed to be associated with tumorigenesis [27,28], SAA may play a role in local inflammation in the microenvironment of the malignant tissue by inducing the production of pro-inflammatory cytokines; tumor necrosis factor-α (TNFα); interleukin-1b (IL-1); and the chemokines CCL1, CCL3, and CCL4 [29-31]. SAA1 also promotes the up-regulation of genes involved in phagocytosis, anti-apoptosis, and tissue remodeling [26]. Second, as it does for human leukocytes [32,33], SAA may induce the adhesion of tumor cells and may enhance tumor cell survival and proliferation, as shown in rheumatoid arthritis [34-36]. Third, SAA may enhance tumor cell invasion and metastatic spread by becoming directly involved in enhancing the activity of matrix degrading enzymes (MMP/TIMP-1) and by increasing TNFα production in and through its association with collagen cleavage in vivo [22,35,36]. Finally, SAA-derived peptides may inhibit tumor cell attachment to extracellular matrix proteins, as shown for T-lymphocytes and platelets [37,38]. Whether any of these possibilities have relevance in the development of human CC requires further investigation.

To the best of our knowledge, this is the first systematic study describing the expression of SAA in human CC tissues. We have demonstrated that SAA mRNA and protein expression levels were high in cervical tumors compared with NNL cervical tissues. In addition, high concentrations of SAA were detected in the sera of CC patients. Given that an HPV screening assay is not in itself sufficient for identifying cervical cancer, some other methods, such as the novel biomarker Lam-5, which is detected in cervical adenocarcinoma [39], and the hTERC amplification test [40], have been reported. Although our data are preliminary and exploratory and require further validation in future studies, they support the hypothesis that SAA may be involved in cervical tumorigenesis and may be a candidate tissue and serum biomarker and perhaps a therapeutic target. Furthermore, combining the new assays described above may constitute a more useful method for monitoring cervical cancer.

However, although our data on the SAA serum levels reveal an obviously increasing trend in patients with NNL cervical diseases, CIN, and stage I and II cervical carcinomas, the NNL and CIN groups and the stage I and II carcinoma groups lack statistical significance. Therefore, larger studies that include more patients would be necessary to confirm this hypothesis that SAA levels increase with increasing disease progression. Additionally, which up- or downstream signaling pathways could be useful for CC treatment remains unknown. Future studies that investigate the specific mechanism of SAA secretion in CC cells may help clarify this issue.

## Abbreviations

SAA: Serum amyloid A; aSAA: Acute phase SAAs; CIN: Cervical intraepithelial neoplasias; ELISA: Enzyme-linked immunosorbent assay; FIGO: Federation International of Gynecology and Obstetrics; HPV: Human papillomavirus; IHC: Immunohistochemistry; IL-1: Interleukin-1b; MMPs: Matrix metalloproteinases; NAC: Indicate neoadjuvant chemotherapy; NNL: Non neoplastic lesion; OD: Optical density; PCR: RT-Real-time polymerase chain reaction; SCC: Squamous cell carcinoma; SD: Standard deviation; TNFα: Tumor necrosis factor-α; ΔCt: The differencein threshold cycles for target.

## Competing interests

We declare that there is no involved conflict of interest.

## Authors’ contributions

YR carried out the histopathology and biochemistry studies, acquired, analysed and interpreted the data and drafted the manuscript. HW was involved in revising the manuscript critically for important intellectual content. DL conceived of the study, and participated in its design and coordination and helped to draft the manuscript. XY and XC were involved in the statistical analysis. JP and QH participated in the collection of tissue and serum specimens. GS has made substantial contributions to conception and design of the manuscript. SL gave final approval of the version to be published. All authors read and approved the final manuscript.
